# The development and validation of a patient-reported outcome measure to assess financial hardship among older cancer survivors in China: hardship and recovery with distress survey

**DOI:** 10.3389/fonc.2023.1151465

**Published:** 2023-04-21

**Authors:** Li Liu, Aihua Zhang, Mingzhu Su, Xiaojie Sun, Di Shao, Joyce Cheng, Nengliang (Aaron) Yao

**Affiliations:** ^1^ Centre for Health Management and Policy Research, School of Public Health, Cheeloo College of Medicine, Shandong University, Jinan, Shandong, China; ^2^ National Health Commission Key Lab of Health Economics and Policy Research, Shandong University, Jinan, Shandong, China; ^3^ School of Nursing and Rehabilitation, Cheeloo College of Medicine, Shandong University, Jinan, Shandong, China; ^4^ School of Nursing, Shandong First Medical University, Shandong Academy of Medical Sciences, Taian, Shandong, China; ^5^ School of Medicine, Johns Hopkins University, Baltimore, MD, United States; ^6^ School of Medicine, University of Virginia, Charlottesville, VA, United States

**Keywords:** older cancer survivors, health outcome, patient-reported outcome measure, financial hardship, financial toxicity

## Abstract

**Background:**

Financial hardship has been described as a patient’s economic experiencefollowing cancer-related treatment. Standardized patient-reported outcome measures(PROM) to assess this distress has not been well-studied, especially among older cancer survivors.

**Objective:**

The aim of this study was to develop and validate PROM for assessing the financial hardship of older cancer survivors in China.

**Methods:**

Items were generated using qualitative interviews and literature review. Items were screened based on Delphi expert consultation and patients’ opinions. Item response theory (IRT) and classical test theory (CTT) were used to help reduce items. Retained items formed a pilot instrument that was subjected to psychometric testing. A cut-off score for the new instrument for predicting poor quality of life was identified by receiver operating characteristic (ROC) analysis.

**Results:**

Qualitative interviews and literature review generated 135 items, which were reduced to 60 items because of redundancy. Following Delphi expert consultation and patients’ evaluation, 24 items with high importance were extracted. Sixteen items were selected due to satisfactory statistical analysis based on CTT and IRT. Ten items were retained and comprised 2 domains after loadings in exploratory factor analysis (EFA). Internal consistency was satisfactory (α = 0.838). Test-retest reliability was good (intraclass correlation, 0.909). The ROC analysis suggested that the cut-off of 18.5 yielded an acceptable sensitivity and specificity.

**Conclusions:**

The PROM for Hardship and Recovery with Distress Survey (HARDS) consists of 10 items that specifically reflect the experiences of financial hardship among older Chinese cancer survivors, and it also showed good reliability and validity in clinical settings.

## 1 Introduction

Financial hardship is defined as patients often being confronted with negative financial consequences of cancer treatment, which include material hardships (e.g., significant out-of-pocket, loss of income), psychological response measures (e.g., distress, stress due to paying medical bills), and coping behavioral measures (e.g., delaying cancer treatment, skipping medications) (1, 2). Financial hardship has a negative effect on cancer patients’ health-related quality of life (HRQoL) and clinical outcomes. Patients with financial hardship were likely to show cancer-related medication nonadherence; worse overall physical, emotional, and social functioning; and decreased well-being (3, 4). Cancer survivors experienced severe and persistent financial hardship long after a cancer diagnosis and regarded it as one of their prime unmet survivorship demands (5).

Although the near-universal population coverage offered by social insurance in China has reduced the proportion of out-of-pocket spending, cancer therapies may still require substantial expenditures even among those with medical insurance. There are two basic health insurance schemes with different reimbursement proportions covering more than 95% of Chinese people, Urban Employee Basic Medical Insurance (UEBMI) and Urban-Rural Resident Basic Medical Insurance (URRBMI) (6). Generally speaking, UEBMI has a better benefits package and lower out-of-pocket costs than URRBMI (7, 8). However, approximately two-thirds of older adults [more than 60 years or older (9, 10)] participate in URRBMI. Patients covered by URRBMI had lower health care utilization and direct medical costs than those covered by UEBMI but paid higher out-of-pocket costs. Therefore, the URRBMI only provides a low level of medical security for members (11). Compared to the experiences of older patients in Western countries, the financial hardship of Chinese patients has been found to be worse (12, 13). Some older cancer survivors borrowed money because of cancer (12). In the context of Chinese culture, the tradition of filial piety is still prominent, meaning that adult children are expected to provide love, respect, material provisions, and physical care to their parents (14). A prior study found that a majority of older patients had to depend on their children to pay for cancer costs; thus, cancer-related financial hardship extended into children’s families (15). Therefore, cancer-related financial hardship among older adults is an important challenge for the healthcare system and patients’ extended families.

The need for specific instruments to estimate financial hardship has been acknowledged in previous research. In the USA, the Comprehensive Score for financial Toxicity (COST) was developed based on patient-reported outcome measures (PROM), which were validated for measuring financial hardship in cancer patients with advanced cancer and undergoing chemotherapy (16). The Financial Index of Toxicity (FIT) was developed and validated to measure financial hardship for patients with head and neck cancer in Canada (17). The Patient Reported Outcome for Fighting Financial Toxicity of cancer (PROFFIT) was designed for patients undergoing cancer treatment in Italy (18). All of these current instruments were created in relatively wealthy, developed countries in the west (19). In fact, they are not always appropriate for use in China, due to social, economic, and cultural differences between developed and developing countries (20). In particular, older cancer patients have a high risk of occurrence of comorbidities, geriatric syndromes, and disability, which significantly reduced the HRQoL of patients and caused catastrophic expenses (21). In order to alleviate medical economics burdens for older adults with cancer in China, it is essential to gain a thorough understanding of cancer-related financial hardship and its effects.

However, there is no a special instrument to describe the effects of cancer-related financial hardship among older adults in China. This theoretical framework was based on a typology of three broad domains of financial hardship. These three domains cover the following aspects: (i) the material conditions that arise from increased direct and indirect costs, (ii) the psychological response as a result of efforts necessary to cope with the increased costs and(iii) the coping behaviors itself that patients adopt to manage their medical care while experiencing increased expenses (22). The aim of this study was to develop a PROM for assessment of financial hardship among older adults with cancer that captures and integrates the relevant domains of subjective financial distress. The following specific aims guided our study: (1) develop a new measure of financial hardship for older cancer survivors in China; (2) evaluate the reliability and validity of the instrument; (3) validate this new instrument in clinical settings.

## 2 Materials and methods

### 2.1 Ethical approval

The approval for this study was provided by the Ethics Committee of the Centre for Health Management and Policy Research at Shandong University (ECSHCMSDU20200901). Participants who understood the research purposes and provided written informed consent were included. The development and validation of the instrument were performed in accordance with the Consensus-based Standards for the selection of health Measurement Instruments (COSMIN) (23). The size and criteria of the sample was shown in the **Supplemental Table 1**.

### 2.2 Item generation and instrument development

#### 2.2.1 Item generation

The original item pool was constructed through qualitative interviews and literature reviews. We interviewed 21 older cancer survivors, 20 family caregivers, 6 oncologists, and 8 nurses using purposive sampling to explore the experiences of cancer-related financial hardship among cancer survivors, and ensure adequate representation of the conceptual domain. The early qualitative findings of the project were published (24, 25). A literature review was performed through PubMed, Web of Science, and Cochrane using selected keywords such as “financial hardship”, “financial toxicity”, “financial burden”, “financial stress/distress”, “cancer survivor”, “cost of cancer care” and “patient-reported outcome (PRO)” to extract published items related to measuring financial hardship after cancer treatment. First, the items the research team members jointly analyzed, while checking for redundancy, overlapping content, and ambiguous language. Second, the items were discussed with anyone with a different view until consensus was reached through consolidation, reflection, and theoretical thinking. Finally, if discrepancies could not be resolved, all team members held weekly online meetings to discuss the pending items and further voting produced the final result.

#### 2.2.2 Item importance evaluation

A Delphi method was used to evaluate the feasibility and importance of the items in the pool. A questionnaire was emailed to 23 experts representing diverse expertise in oncology-related fields (e.g., oncology, nursing, psychology, health economics). Experts were asked to rate each item in the initial pool according to (a) rationality and specificity; (b) feasibility and representativeness of implementation into clinical practice (26). Each rating was made on a 0 (low) to 10 (high) scale. To reinforce the understanding of the link between financial hardship and item content, we also invited 40 patients for the importance of the items and cognitive test. We collapsed the options to “important (assign it the value of 1)” and “not important (assign it the value of 0)” to define whether items were important and the mean values of importance scores were calculated. Finally, the items with mean value ≥0.6 (i.e., support rate ≥ 60%) were retained (16).

#### 2.2.3 Item analysis

Older survivors who had received any cancer treatment for at least one consecutive month were included in this step. The item analysis based on Classical Test Theory (CTT) mainly included: critical ratio (CR), reliability analysis, option selection rate analysis, and correlation coefficient. Item Response Theory (IRT) was used to explore the ability and response at every level among the participants. Specifically, two-parametric logistic regression model analysis were used for dichotomous variables and five-point Likert items were analyzed by the Graded Response Model (GRM). We also performed each item’s discriminability and difficulty, item characteristic curve (ICC), item information function (IIF), and scale information function (SIF) to assess internal validity of the instrument. We deleted items with unsatisfactory indicators after group meeting. In Exploratory Factor Analysis (EFA), the factor structure used principal axis factoring analysis. Factors with eigenvalues greater than 1.0 were retained using the Kaiser–Guttman principle. We used a scree plot and parallel analysis to examine the retained factors. Factor loadings over 0.5 showed theoretical and practical significance.

### 2.3 Instrument validation

#### 2.3.1 Reliability and validity

Internal reliability of the multi-item instrument was assessed by analyzing inter-item correlations and using Cronbach’s α coefficient adjusted by the number of items. An estimate of Cronbach’s α >0.70 was considered to indicate acceptability (17). To analyze the test-retest reliability, the intraclass correlation coefficient (ICC) was estimated by repeating the questionnaire between the patients’ first survey and approximately 2 weeks later, with an ideal level of ICC ≥0.7 (27). Confirmatory factor analysis (CFA) was used to evaluate the overall structural validity of the instrument with criteria for a good model fit identified as: Root Mean Square Error of Approximation (RMSEA) ≤0.080, a value of ≥0.960 for Comparative Fit Index (CFI), and Tucker–Lewis Index (TLI), Goodness of Fit Index (GFI)≥0.900 (27). Convergent validity was evaluated by using the average variance extracted (AVE) and composite reliability (CR). Discriminant validity and criterion validity of the new instrument was also determined.

#### 2.3.2 Identifying a cut-off score

Cut-off scores were determined using receiver operating characteristic (ROC) analyses, which produce a comprehensive assessment of diagnostic values for sensitivity and specificity. Since the ROC curve plots the relationship between the true positive rate (sensitivity) and the false positive rate (1-specificity), it can help in selecting a value with the best predictive power. We assessed the accuracy of this prediction by the area under the ROC curve (AUC). If a value of closer to 1.0 indicates more perfect accuracy, a value ≤ 0.5 shows lower accuracy. The cut-off score of the new instrument was determined by ROC analysis based on its discriminatory ability to predict the first quartile of the PROM 10-item Global Health Scale in measuring for HRQoL (28). With poor quality of life as the health outcome, the financial hardship score was divided into higher and lower financial hardship. Finally, a multivariable logistic regression model was used to examine the relationship between cancer survivors’ characteristics and higher financial hardship. Independent variables included sociodemographic and cancer characteristics.

### 2.4 Statistical analysis

IRT analysis was performed using R programs to select items. The CTT analyses and EFA were conducted in SPSS 21.0. A parallel analysis was performed in MonteCarlo PCA to determine the most appropriate number of factors to extract. The data was submitted for further CFA using AMOS 24.0 with the maximum likelihood method. The characteristics of the participants such as frequency, percentage, means, and standard deviation were analyzed using descriptive statistics. Independent variables with a P value <0.05 on univariate analysis were entered into a multivariate logistic regression model analysis by adopting the stepwise method. All tests were 2-sided, and a P value < 0.05 was considered statistically significant. ROC and Logistic regression analyses were performed using SAS 9.4. All analyses were performed in 2021.

## 3 Results

### 3.1 Item generation and instrument development

#### 3.1.1 Item generation

**Figure 1:**
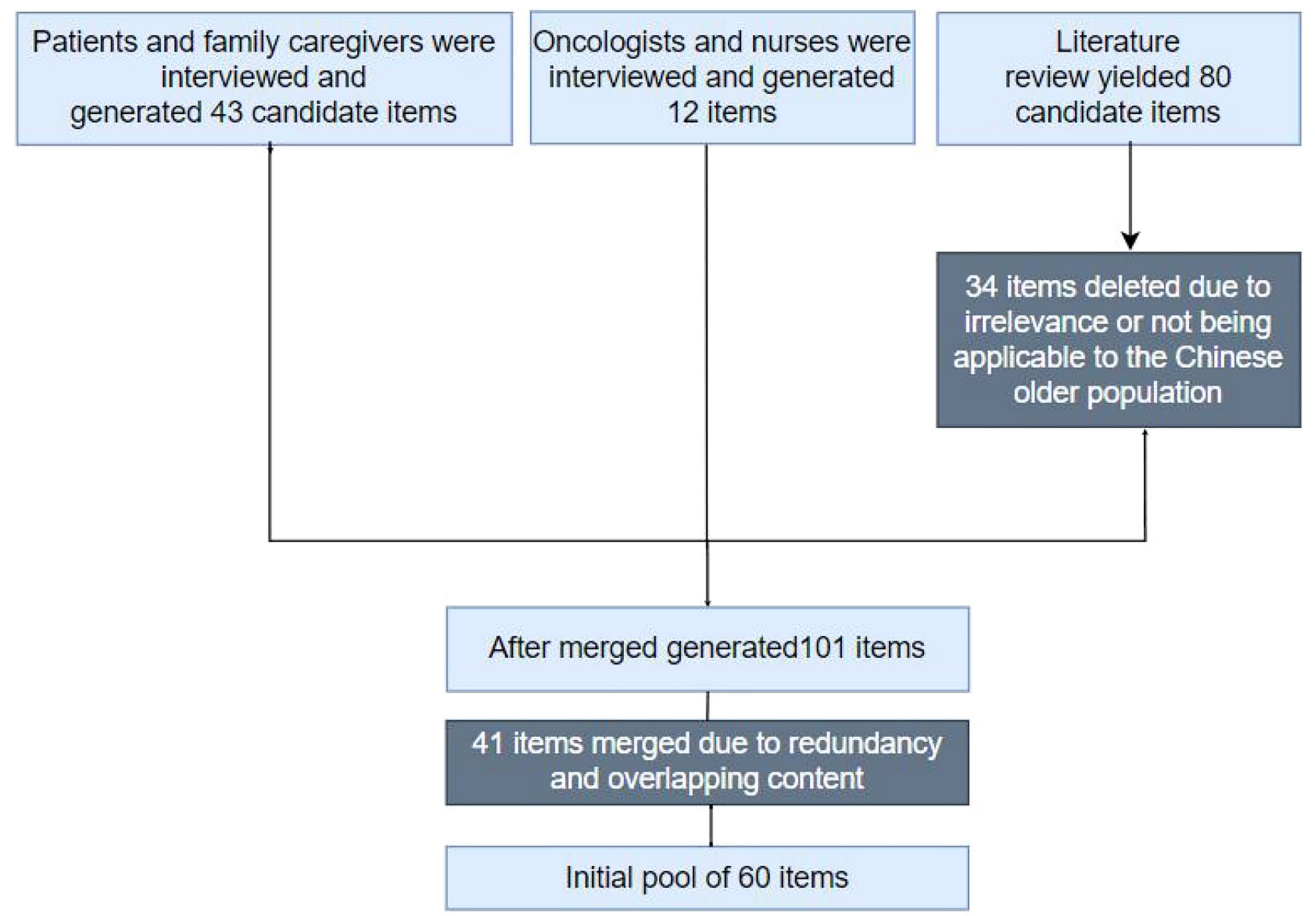
Initial items pool.

Literature review yielded 80 candidate items. An additional 43 candidate items were generated by interviews with 21 survivors and 20 family caregivers, while an additional 12 items were generated from feedback from 6 oncologists and 8 nurses. These 135 items were reduced to 60 by the investigators because of redundancy and overlapping content (see **Figure 1**).

#### 3.1.2 Item importance analysis

Two Delphi rounds were conducted. The response rate of the questionnaire was 82.6% (19/23) in round 1, and 89.5% (17/19) in round 2. In the 2-round Delphi methods, the experts’ authority coefficients were more than 0.700; they were 0.800 in round 1 and 0.897 in round 2. Finally, 36 items were deleted and 5 items were added by experts. In total, 29 items were retained. Subsequently, the important support rate of items from the 40 patients ranged from 17.5% to 100%. 5 items were excluded by an important support rate of <60%; finally, 24 items were retained (see the **Supplemental Table 2**).

#### 3.1.3 Item reduction

The IRT-based item analysis showed good parameter of discrimination and difficulty among majority of items; ICC, IIF, and SIF were well distributed. The CTT based analysis item CR suggested that the majority of items had good discrimination. The correlation coefficient method indicated that most items had a good correlation with the total score, and some items had a strong correlation (≥0.70, P<0.01). Cronbach’s α coefficient indicated that the correlation coefficients of a few items (item 6, item 7, and item 8) were all less than 0.350 after correction, and the Cronbach’s alpha if item deleted (CAID) values increased. Finally, 8 items were removed from the items pool following the criteria mentioned in the methods section above (see **Table 1**).

**Table 1:** Item reduction results using the IRT and CTT.

Item	IRT		CTT					Outcome
	a	b	CITC	Effectiveness*	IIC	TIC	CR	
Item 6	0.517	0.390	0.336	45.4		0.397	<0.001	√
Item 7	1.165	-2.206	0.324	10.7		0.382	<0.001	√
Item 8	0.400	-9.975	0.014	2.0		0.047	>0.050	×
Item 9	1.003	4.017	0.481	3.4		0.473	<0.001	×
Item 10	0.923	4.212	0.375	3.4		0.346	<0.001	×
Item 12	2.361	1.874	0.755	11.7	>0.7	0.738	<0.001	×
Item 14	2.942	1.880	0.750	7.3		0.746	<0.001	√
Item 16	1.976	2.048	0.661	6.8	>0.7	0.626	<0.001	×
Item 20	3.199	1.490	0.762	7.3	>0.7	0.776	<0.001	×
Item 23	3.857	1.676	0.762	8.8	>0.7	0.749	<0.001	×
Item 24	3.824	1.715	0.734	8.8	>0.7	0.714	<0.001	×

“√” represented the selected item;

“×” indicated the item considered to be deleted;

*One of the response options is less than 10%;

a, discrimination parameter, an item should have a discrimination value greater than 0.35;

b, difficulty parameters, the difficulty values should range from −3 to 3;

Abbreviations: CITC, corrected item-total correlation;

IIC, interitem correlation, if the IIC ≥0.7, compared to the two items’ importance score in methods 2.2.2, the item with a lower score was deleted;

TIC, total-item correlation;

CR, critical ratio.

#### 3.1.4 Exploratory factor analysis

**Figure 2:**
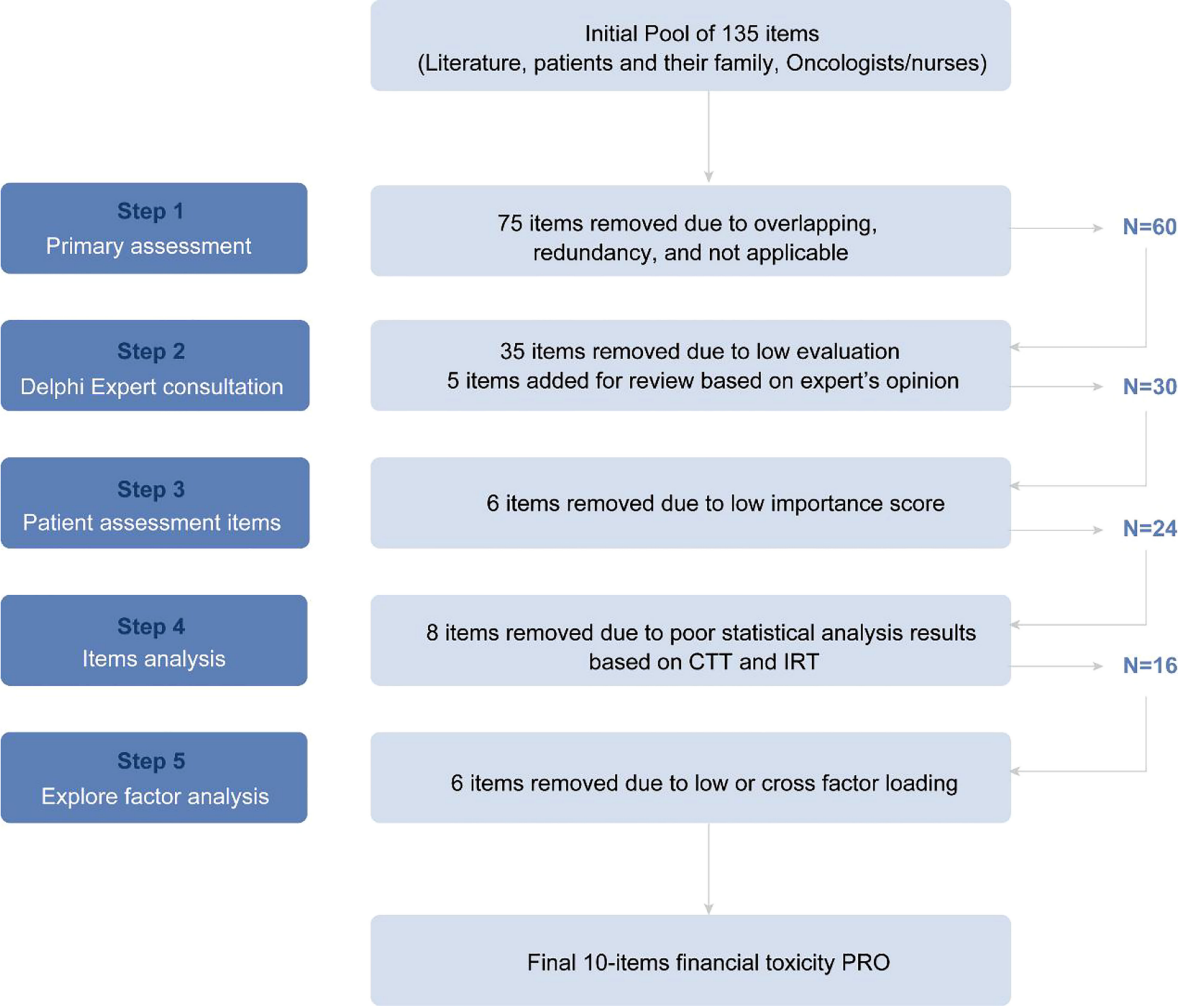
Flow chart of items inclusion and deletion.

The parallel analysis and scree plot results show two factors would be extracted. Six items were deleted because they did not load on either of the extracted factors. The EFA was then conducted again on the remaining ten items. The Kaiser-Meyer-Oklin value was 0.842, and Bartlett’s spherical test P< 0.5. Two factors explained 56% of the variance and were named “subjective financial distress” (items 14,15, 18, 19, and 22) and “objective medical burden” (items 1, 2, 3, 4, and 5) (see **Table 2**). This new 10-item version was then developed, which was named the “Hardship And Recovery with Distress Survey” (HARDS) (see **Supplemental Table 4**). The total score range was from 10 (highest financial hardship) to 50 (lowest financial hardship). **Figure 2** summarizes the adopted stepwise approach.

**Table 2:** Factor loadings of the remaining 10-item HARD using EFA.

Item	Extracted factors		Communality
	Subjective financial distress	Objective medical burden	
Item 1	-0.094	**0.640**	0.354
Item 2	-0.018	**0.773**	0.583
Item 3	0.016	**0.781**	0.623
Item 4	0.172	**0.448**	0.312
Item 5	0.016	**0.548**	0.310
Item 14	**0.797**	0.042	0.673
Item 15	**0.884**	-0.123	0.680
Item 18	**0.662**	-0.002	0.437
Item 19	**0.446**	-0.011	0.194
Item 22	**0.544**	0.206	0.459
Percent variance (%)	40.8	15.3	56.1
Factors correlation		0.534	

Factor loadings of ≥ 0.4.in bold.

### 3.2 Instrument validation

#### 3.2.1 Reliability analysis

The Cronbach’s α for the 10-item instrument was 0.838. The Cronbach’s α for factor 1 and factor 2 were comparable at 0.856 and 0.865, respectively. The test-retest reliability of the measure was 0.909 from a sample size of 23 patients who were assessed twice within 14 days. The result of CITC and CAID were shown **Table 3**.

**Table 3:** Results of the reliability analysis.

Item		CITC	CAID
HARD1	I couldn’t afford the costs of my cancer treatments and care.	0.465	0.834
HARD2	I don’t have enough income, savings, or retirement pension to cover my treatment costs.	0.489	0.832
HARD3	I rely on my children to pay for my medical costs.	0.537	0.830
HARD4	Due to cancer treatment and related long-term impacts on my daily life, I had to borrow money or was in debt.	0.441	0.835
HARD5	I used up all my savings for my cancer treatment.	0.325	0.839
HARD6	I worry that my cancer treatment will affect my family’s financial stability.	0.729	0.801
HARD7	I worried about the loss of both my life and money at the end of my cancer treatment.	0.699	0.805
HARD8	If the expected medical cost is higher than I can afford, I would give up the treatment.	0.614	0.817
HARD9	Due to financial reasons, I would choose the medications covered by medical insurance.	0.677	0.809
HARD10	I reduced spending on basics like food or clothing because of the costs of my cancer care.	0.629	0.813

item 1=HARD1, item 2= HARD 2, item 3= HARD 3, item 4= HARD 4, item 5= HARD 5, item 14= HARD 6, item 15= HARD 7, item 18= HARD 8, item 19= HARD 9, item 22= HARD 10; CITC, corrected item-total correlation; CAID, Cronbach’s alpha if item deleted.

#### 3.2.2 Validity analysis

**Figure 3:**
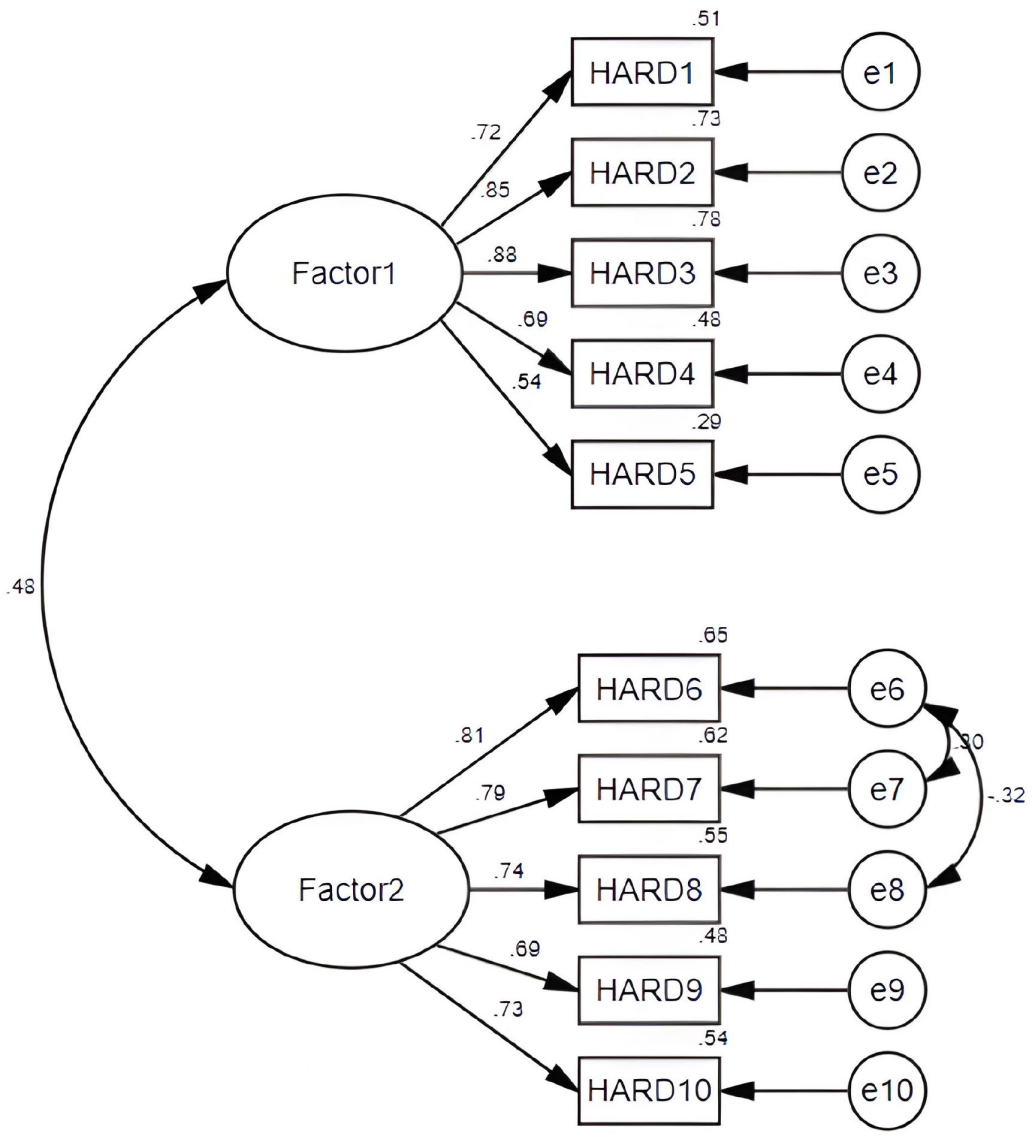
A two-factor model for the HARD from confirmatory factor analysis.

The results of CFA showed that the tool had good structural validity; the loading of each factor ranged from 0.548 to 0.884. The corrected model fit indices were ideal (RMSEA=0.075, SRMR=0.041, GFI=0.956, CFI=0.964, TLI=0.949**)** (see **Figure 3**). The correlation coefficient between the total score of the COST scale and the HARDS total score of this measuring tool was 0.523 (P<0.01), which indicated that the criterion validity of the HARDS was satisfactory. The AVE of the two factors were 0.555 and 0.558, respectively. The CR value of the two factors were 0.859 and 0.860, respectively.

#### 3.2.3 Cut-off analysis

The mean score of the HARDS for financial hardship was 20.4 (standard deviation = 6.4). The ROC analysis results suggested this cut-off score of 18.5 could provide a balance between acceptable levels of sensitivity (0.64) and specificity (0.59). When the sample was stratified based on this cut-off score, 42% of samples were defined as having higher financial hardship.

#### 3.2.4 Results of the multivariate regression analysis

The influencing factors of high financial hardship included socioeconomic status (i.e., employment, household income, education, and medical insurance type), social support, loneliness, frailty status, cancer site, out-of-pocket costs, and medical decision-making patterns. Higher socioeconomic status of patients was associated with lower financial hardship (OR=0.427, 95%CI:0.326~0.560). Samples with frailty had a higher probability (OR=1.817) of financial hardship than those who were non-frail (see **Table 4**).

**Table 4:** Results of Logistic regression analysis (Reference: Lower financial hardship).

Variables	β	OR (95%CI)	P-value
Socioeconomic status	-0.851	0.427 (0.326, 0.560)	<0.001
Social support	-0.023	0.977 (0.958, 0.997)	0.026
Loneliness (Reference: No)			
Yes	0.394	2.200 (1.024, 4.726)	0.020
Frailty (Reference: No)			
Yes	0.299	1.817 (1.098, 3.007)	0.026
Cancer site (Reference: Lung)			
Esophageal and Stomach	-0.500	0.908 (0.467, 1.765)	0.061
Colorectum	0.267	1.995 (0.783, 4.881)	0.460
Liver and gallbladder	0.796	3.302 (1.504, 7.251)	0.011
Other	-0.156	1.281 (0.651, 2.520)	0.562
Out-of-pocket (/10,000 CNY)	0.050	1.051 (1.021, 1.081)	<0.001
Medical decision making (Reference: Patients)			
Family	-0.546	0.339 (0.135, 0.848)	0.014
Shared	0.254	0.754 (0.316, 1.801)	0.197
Oncologist	-0.245	0.458 (0.169, 1.242)	0.349

Socioeconomic status was defined by education level, occupation, annual household income, and health insurance. It was determined as a continuous variable by principal component analysis;

The Multidimensional Scale of Perceived Social Support (MSPSS) was used to assess social support;

The Groningen Frailty Indicator Scale (GFI) was used to assess the frailty level;

10,000 CNY was approximately US $1,433 as of December 31, 2021.

OR, odds ratio; CI, confidence interval; β, effect estimate.

## 4 Discussion

### 4.1 Main findings

The HARDS, containing 10 items, is a new tool for measuring cancer related financial hardship for older patients in China, that takes about 5 minutes per patient to measure. The HARDS captures the subjective financial distress and objective medical burden. The HARDS based on PROM can reflect the specificity of older cancer survivors’ experiences. The collection and use of PROM such as the HARDS can help with medical decision-making, early identification of financial hardships, and improvements to HRQoL and prognosis. In this study, we used the COSMIN checklist to evaluate the methodological quality of studies on the measurement properties of PROM measuring financial hardship for older cancer survivors (29). And the validity and reliability of the HARDS as a screening tool for financial hardship have been tested. We also determined the cut-off score that predicted a poor outcome for HRQoL, as well as features that characterize older survivors with a high level of financial hardship.

Current instruments measuring cancer related financial hardship include the FIT (17), the COST (30), and the Breast Cancer Finances Survey (BCFS) (30, 31). The FIT was designed specifically for head and neck cancer, the COST was designed for patients with advanced cancer, and the BCFS was designed exclusively for breast cancer patients. Applicability of these instruments to other cancer stages and sites may be limited. To our knowledge, the COST is currently the most commonly used validated instrument to measure financial hardship in cancer survivors (31, 32). However, the COST measure has only one family item which is a summary statement (27); thus, the financial hardship on families has not been fully taken into consideration. In this study, cancer-related financial worries and stress among older adults extended into their families, especially those of their adult children. Our instrument assessed financial hardships from the perspectives of both an individual and their family. Therefore, the HARDS captures the family’s financial situation and covers material factors, psychological measures, and coping strategies to comprehensively measure financial hardship.

Like other studies that have developed and validated measures of financial toxicity, our study also uses the COST as the gold standard for criterion validity (17, 18, 33). For example, factor analysis and item reduction were performed on the patients as validity testing. The instrument demonstrated reasonably good psychometric properties, which provide useful information for practical applications. Thus, HARDS is a valid, reliable tool. But one of the job-related items of COST “I am concerned about keeping my job and my income” might be less sensitive to older survivors. In China, the older population in rural and urban areas aged 60 were 175 million and 75 million, respectively, and nearly 70% of older people lived in rural areas (34). Rural residents lack pension support and expect to work in agriculture-related activities until relatively late in their lives. Furthermore, older adults in urban areas usually have retired, so their job and salary were rarely affected due to cancer treatment. Despite deleting this job-related item, the rest of 10 items retained were representative of the COST with a score ranging from 0 to 40. The results still indicated that the newly developed HARDS correlated well with the modified COST.

This study also determined a proposed cut-off score for the HARDS measure. The cut-off score predicted an adverse outcome for HRQoL and categorized the level of high or low financial hardship. Forty-two percent of the patients had a high level of financial hardship in our study. A prior study indicated close to 20% of older adults with advanced cancer experience financial hardship in USA (35). In the USA, most respondents were aged 65, and older adults often had Medicare, while lower-income people were enrolled in Medicaid (36). These insurance programs help them pay for medical services, including hospitalization, prescription drugs, home health care, and hospice care. However, China has implemented a basic medical insurance system, in which UEBMI is mandatory for employees in urban areas, while unemployed residents in urban areas and rural residents are covered by the URRBMI (37). In China, most of the older patients are farmers, and they are a relatively disadvantaged population with low incomes. A previous study indicated that older cancer survivors from rural areas have to bear higher hidden costs of transportation and rent for their homes (27). Moreover, rural residents are covered by URRBMI, which has a lower reimbursement ratio than UEBMI. Thus, the cancer-related financial hardship prevalence in rural patients is higher than in those with pension support and prior non-agricultural employment (38). A considerable proportion of older patients still struggle against financial hardship despite the availability of basic health insurance. There is a gap that needs to be addressed between financial hardship and government assistance (39, 40). Therefore, these medical insurance policies need to be constantly improved to alleviate the burden of cancer-related costs.

The strengths of our study included the integration of qualitative interviews with quantitative findings, and the inclusion of a broad stakeholder group with experts from a diverse, yet significant group of oncology-related fields, patients, and their families. This study shows a comprehensive understanding of older cancer survivors and their family members’ financial hardship.

### 4.2 Clinical implications

This study is an original study in the field of cancer survivorship in China that provides evidence for improving the quality of cancer care. The incorporation of financial toxicity assessments into observational research will ensure a patient centered foundation in the evaluation of financial distress, as HARDS is a quick and reproducible measurement that could be used in clinical practice to identify patients early who may be at risk of financial hardship and may benefit most from intervention. Oncology providers (oncologists and nurses) are important agents in patient cancer care experiences, discussions about financial toxicity of cancer care should be initiated by an informed oncologist and managed by the entire healthcare team. The collection and use of the HARDS can help with enhancing shared decision-making between oncologists and patients to reduce costs. Long-term solutions must include policy shifts involving how we set and negotiate anti-cancer prices and insure patients. The HARDS may help increase awareness of patient financial distress and cancer treatment cost sparking discussions among health policy makers and other stakeholders to develop multidisciplinary strategies for mitigating financial toxicity.

### 4.3 Study limitations

Several potential limitations should be considered in interpreting the results of the study. First, the study findings might not be representative of all older cancer patients, as this study did not include individuals who were not admitted to the hospital and did not receive treatment due to severe financial difficulties. Therefore, the level of financial hardship in older populations may be underestimated. Second, the financial toxicity of PROM in China may differ from older cancer survivors in other countries due to social and cultural differences, so the extrapolation of the instrument may be limited. It needs cross-cultural validation and adaption in other eastern countries. Third, this study used a cross-sectional survey in the instrument validation stage, but the trajectory of cancer and medical treatment for survivors is complicated and long-term; thus, a prospective study is needed to determine how financial toxicity changes over time.

## 5 Conclusions

In this study, we report the development and validation of the HARDS to measure financial hardship among older cancer survivors in China. This study found that poor quality of life was associated with a higher level of financial hardship, and the severity cut-off score of the new instrument was obtained. Finally, we also identified several influencing factors on higher financial hardship, such as low socioeconomic status, poor social support, loneliness, frailty, high out-of-pocket costs, and more.

## Data availability statement

The original contributions presented in the study are included in the article/**Supplementary Material**. Further inquiries can be directed to the corresponding author.

## Author contributions

LL: Conceptualization, data curation, software, writing-original draft, and writing–review, and editing. AZ: Writing, data curation, review, software, and editing. MS: Conceptualization, methodology, formal analysis, and writing–review, and editing. XS: Writing–review, and editing. DS: Writing–review, and editing. JC: Writing–review, and editing. NY: Supervision, and writing–review, and editing. All authors contributed to the article and approved the submitted version.
