# Supplementary material for: The development and validation of a patient-reported outcome measure to assess financial hardship among older cancer survivors in China: hardship and recovery with distress survey
Source: Front Oncol. 2023 Apr 21;13:1151465. doi: 10.3389/fonc.2023.1151465 (PMC10162643; doi:10.3389/fonc.2023.1151465)
Supplement: Supplementary file 1 [file DataSheet_1.docx]

Supplemental Table 1 Summary table of implemented tasks

| **Task** | **Instrument Development** | | | **Instrument Validation** | |
| --- | --- | --- | --- | --- | --- |
| **Title** | Item generation | Item importance | Item reduction | Reliability and validity | Identify a cut-off score |
| **Actions** | (1) Literature review.  (2) Qualitative interview.  (3) Group discussions. | (1) Delphi expert consultation.  (2) Importance analysis with patients. | (1) Item response theory.  (2) Classical test theory.  (3) Factor analysis | (1) Reliability test.  (2) Validity test. | (3) Receiver operating  characteristic analysis.  (4) Multivariable logistic regression  Analysis. |
| **Sample**  **involved** | (2) 21 older adults with cancer and 20 family caregivers; 6 oncologists and 8 nurses. | (1) 23 experts.  (2) 40 older adults with cancer. | (1) (2) (3) 205 older adults with cancer. | (1) (2) (3) (4) 518 older adults with cancer. | |
| **Inclusion criteria** | **Patients included:**  (a) a diagnosis of cancer;  (b) awareness of their cancer diagnosis;  (c) being at least 60 years old at the time of their cancer diagnosis;  (d) having cancer treatment ongoing (at least three months post-diagnosis) or having completed initial treatment;  (e) ability to give written informed consent.  **Caregivers included:**  (a) mainly responsible for managing patients' finances;  (b) providing significant care;  (c) being aware of patients’ cancer diagnoses.  **Oncologists and nurses included:**  (a) licensed health care providers in China;  (b) worked in the oncology department for at least one year;  (c) have treated or nursed older patients with cancer. | **Experts included:**  (a) diverse expertise in oncology-related fields (e.g., oncology, nursing, psychology, health economics);  (b) intermediate and above titles;  (c) at least five years of working experience.  **Patients included**：  (a) a diagnosis of cancer;  (b) being at least 60 years old at the time of their cancer diagnosis;  (c) self-reported financial difficulties due to cancer treatment;  (d) having cancer treatment ongoing (at least three months post-diagnosis) or having completed initial treatment. | **Patients included:**  (a) a diagnosis of cancer;  (b) awareness of their cancer diagnosis;  (c) being at least 60 years old at the time of their cancer diagnosis;  (d) having cancer treatment ongoing (at least one-month post diagnosis) or having completed initial treatment;  (e) ability to give written informed consent. | | |
| **Exclusion criteria** | **Patients excluded:** (a) cognitive deficits; (b) mental illness; (c) language problems. | | | | |
|  | **Oncologists and nurses excluded:** currently practicing, advancing, or training in the hospital. | | | | |
|  | **Experts included:** not enough time to complete the survey. | | | | |

| Supplemental Table 2 24 items pool after importance evaluation | |
| --- | --- |
| 1 | I couldn’t afford the costs of my cancer treatments and care. |
| 2 | I don’t have enough income, savings, or retirement pension to cover my treatment costs. |
| 3 | I rely on my children to pay for my medical costs. |
| 4 | Due to cancer treatment and related long-term impacts on my daily life, I had to borrow money or was in debt. |
| 5 | I used up all my savings for my cancer treatment. |
| 6 | My children had to increase their number of leave requests and shifts to take care of me. |
| 7 | I had to delay, give up, or adjust my treatment plans due to the cost of treatment. |
| 8 | I used to apply for medical aid or clinical trials to reduce the cost of my cancer treatment. |
| 9 | I think the reimbursement ratio for cancer treatment is not high enough. |
| 10 | I think the prices of out-of-pocket anti-cancer drugs are too high. |
| 11 | My financial situation is under my control. |
| 12 | At my age, I feel helpless to pay for cancer-related costs. |
| 13 | I worry about my ability to pay off my debts after cancer treatment. |
| 14 | I worry that my cancer treatment will affect my family’s financial stability. |
| 15 | I worried about the loss of both my life and money at the end of my cancer treatment. |
| 16 | I worried that the costs of my cancer treatment will put a significant financial burden on my children. |
| 17 | I had asked the doctor to replace the optimal treatment plan with an economical plan due to financial strains. |
| 18 | If the expected medical cost is higher than I can afford, I would give up the treatment. |
| 19 | Due to financial reasons, I would choose the medications covered by medical insurance. |
| 20 | The treatment of my illness put financial strains on me, even though medical insurance covered part of the expenses. |
| 21 | I can afford my monthly expenses. |
| 22 | I reduced spending on basics like food or clothing because of the costs of my cancer care. |
| 23 | I feel financially stressed due to my treatment. |
| 24 | I worry about the high cost of cancer treatment. |

| Supplemental Table 3 The Hardship And Recovery with Distress Survey | | | | | | |
| --- | --- | --- | --- | --- | --- | --- |
| **Items** | | **Response** | | | | |
| 1 | I was unable to cover the costs of my cancer care. | Yes (1) | | | No (5) | |
| 2 | I didn’t have enough money in savings, retirement, or assets to cover the costs of my treatment. | Yes (1) | | | No (5) | |
| 3 | I had no choice but to rely on my children to pay for medical costs. | Yes (1) | | | No (5) | |
| 4 | I've had to borrow money or go into debt because of the costs of my cancer care. | Yes (1) | | | No (5) | |
| 5 | The costs of my cancer care ate up my money. | Yes (1) | | | No (5) | |
| 6 | I worried about my family’s financial stability because of the costs of my cancer care. | Strongly agree (1) | Agree (2) | Moderately (3) | Disagree (4) | Strongly disagree (5) |
| 7 | I worried about the loss of both my life and money in the future as a result of my cancer care. | Strongly agree (1) | Agree (2) | Moderately (3) | Disagree (4) | Strongly disagree (5) |
| 8 | If medical costs are too much for my present financial situation, I would give up my treatment. | Strongly agree (1) | Agree (2) | Moderately (3) | Disagree (4) | Strongly disagree (5) |
| 9 | I have reduced spending on basics like food or clothing because of the costs of my cancer care. | Very much (1) | Quite (2) | Somewhat (3) | A little bit (4) | Not at all (5) |
| 10 | I would choose the medications for my cancer care covered by my insurance. | Strongly agree (1) | Agree (2) | Moderately (3) | Disagree (4) | Strongly disagree (5) |

*Note:* The total score range was from 10 (highest financial toxicity) to 50 (lowest financial toxicity).

| Supplemental Table 4 Characteristics of older survivors with cancer | | |
| --- | --- | --- |
| **Variables** | **Participant characteristics in item reduction stage (n=205)** | **Participant characteristics in instrument validation stage (n=518)** |
| **Age group,（years）** |  |  |
| 60-64 | 59 (28.8) | 143 (27.6) |
| 65-69 | 79 (38.5) | 182 (35.1) |
| 70-74 | 44 (21.5) | 121 (23.4) |
| 75-80 | 17 (8.2) | 52 (10.0) |
| ≥80 | 6 (3.0) | 20 (3.9) |
| **Sex** |  |  |
| Male | 131 (63.6) | 309 (60.1) |
| Female | 74 (35.4) | 205 (39.9) |
| **Marital status** |  |  |
| Married | 198 (96.1) | 489 (94.4) |
| Other | 7 (3.9) | 29 (5.6) |
| **Monthly family income^a^** |  |  |
| ＜1000 CNY | 50 (24.3) | 131 (26.3) |
| 1000-2999 CNY | 59 (28.8) | 105 (21.1) |
| 3000-4999 CNY | 53 (25.9) | 138 (27.7) |
| 5000-9999 CNY | 30 (14.6) | 80 (16.1) |
| ≥10000 CNY | 13 (6.3) | 44 (8.8) |
| **Job** | | |
| Farmer or part-time | 87 (42.4) | 232 (44.8) |
| Unemployed | 60 (29.3) | 127 (24.5) |
| Retired | 34 (16.5) | 80 (15.4) |
| Other | 24 (11.7) | 79 (15.3) |
| **Education** |  |  |
| Elementary or less | 95 (46.3) | 230 (44.4) |
| Secondary school | 58 (28.3) | 131 (25.3) |
| High school or higher | 52 (25.4) | 157 (30.3) |
| **Health insurance type** |  |  |
| URRBMI | 114 (55.6) | 295 (57.2) |
| UEBMI | 91 (44.4) | 221 (42.8) |
| **Cancer site** |  |  |
| Lung | 94 (45.9) | 230 (46.2) |
| Upper digestive | 35 (17.1) | 90 (18.1) |
| Colorectal | 11 (5.4) | 39 (7.8) |
| Liver and gallbladder | 27 (13.2) | 55 (11.0) |
| Others | 38 (18.5) | 84 (16.9) |
| **Cancer stage** |  |  |
| 0-I | 22 (10.7) | 65 (12.5) |
| II | 48 (23.4) | 117 (22.6) |
| III-IV | 70 (34.1) | 184 (35.5) |
| Missing | 65 (31.7) | 152 (29.3) |
| **Time since cancer diagnosis** | | |
| ≤1 y | 131 (63.9) | 338 (66.9) |
| >1 y | 74 (36.1) | 167 (33.1) |

Abbreviations: CNY, Chinese yuan; URRBMI, Urban-Rural Resident Basic Medical Insurance; UEBMI, Urban Employee Basic Medical Insurance;

^a^ 10,000 CNY was approximately US $1,433 as of December 31, 2021.


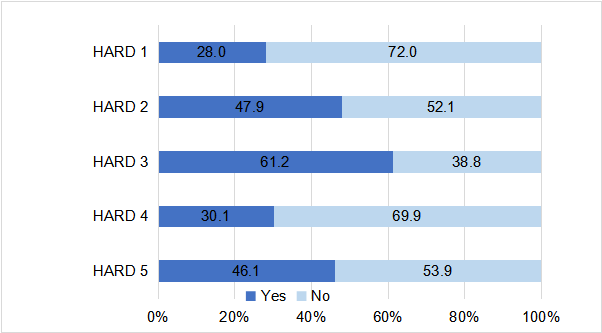


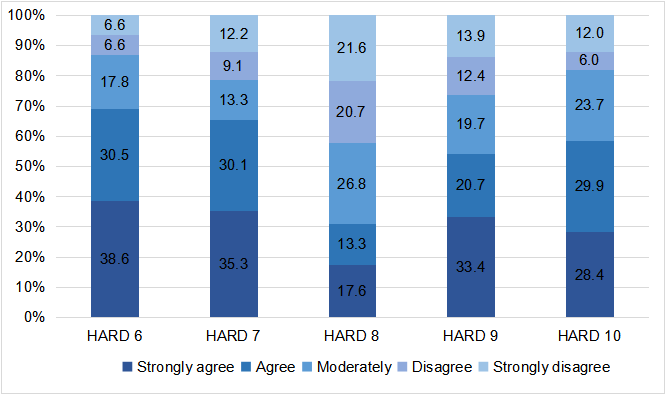


Supplemental Figure The distribution of ten items in HARD among respondents (N=518)
